# Notes on External Applications, Bandages, Baths, &c., in the Treatment of Ascites, with Clinical Reports of Cases Treated in the Philadelphia Hospital

**Published:** 1842-04-02

**Authors:** W. W. Gerhard


MEDICAL EXAMINER.

NEW SERIES.

No. 14.] PHILADELPHIA, APRIL 2, 1842. [Vol. I.

*Notes on External Applications, Bandages, Baths, &c., in the Treatment of Ascites, with Clinical Reports of Cases treated in the Philadelphia Hospital.* By W. W. Gerhard, M. D.

(Cases reported by T. L. Walker, M. D.)

There are stages of ascites where no means of treatment can be successful, that is, when the patient is extremely emaciated, the skin very dry and harsh, and the liver cyrrhosed and contracted: the effusion is in such cases extremely large, and when once firmly established, the secretions of fluid, both urine and perspiration, are almost completely suppressed, and permanently replaced by the new exhalation into the peritoneum. In such cases the natural powers are too much exhausted to be again revived, and if we succeed in removing the effused serum by tapping, or powerful purgatives, or diuretics, the patient generally sinks. The operation in this case is never curative, it relieves the excessive dyspnœa, caused by the pressure from the abdomen on the chest, but it does not remove the cause; on the contrary, it is very evident, from observation, that the rapidity of the secretion is in reality augmented, and the peritoneum fills more rapidly than before.

In less advanced cases of ascites the treatment is palliative at first, because the disorder almost never occurs as a primitive affection, but only as the sequel of long continued liver disease, which again in our climate results in most instances from chronic intermittent fever. Tapping in these cases is resorted to by some practitioners; my own impressions are against it, believing that where successful it is unnecessary, while in the majority of cases it has a positively injurious effect. The usual treatment of the disease by purgatives, diuretics, and occasional alteratives, is too well known to require notice. But there are some other remedies which are either overlooked or forgotten, which, though ostensibly less potent, are in reality of more real value in the treatment of ascites, than powerful hydragogues. These are baths and bandages, excellent adjuvants to internal medicines.

The action of repeated baths is very simple; if the function of the skin be not totally destroyed, a warm water bath, or a warm vapour bath will favour or even reproduce perspiration, and check the tendency to internal exhalation which becomes so strong in all dropsical diseases, but especially in ascites. They must be repeated every day, or every second or third day, according to the strength of the patient; if he should become chilly after the bath, they should be discontinued for a time, but may be almost always soon resumed with perfect safety. I have generally preferred the warm water bath to the vapour, as much more agreeable, but the latter may always be prepared by means of a few hoops, and a blanket wet with hot water and wrapped round some heated bricks, while the warm bath is in some places more difficult.

Compression of the abdomen is another valuable remedy ; it is often used irregularly, but I am not aware that it was ever introduced as an efficient agent in the treatment of ascites before it was used by Bricheteau, of the Neckar Hospital. For the last six years I have very frequently employed it ; in very advanced cases, where the distension of the abdomen is enormous, and the serous membranes of the chest contain much water, the bandage can scarcely be borne, from the increased impediment to the respiration. But in such cases all remedies are of little moment, but when there is moderate effusion the inconvenience of the patient is very trifling, and in some respects it is rather agreeable, by keeping up a gentle support for the excessively distended abdomen. A common broad roller may be used for this purpose, or a broad laced bandage, if well made, so as to press equably on all portions of the abdomen. The roller will, of course, become loose after a time, and must be replaced at least once in the twenty-four hours ; twice would be still better. It should be continued until some time after the effusion is completely removed. The usual remedies for the treatment of ascites may be used in conjunction with the bandage.

The following cases, in which all unnecessary details are avoided, will illustrate the practice. All of them were brought before the clinical class.

Case 1st.—P. B., æt. 30, Irishman, of strong constitution, admitted Dec. 14th, 1841. Had several attacks of intermittent fever. In September, during convalescence from the last, was much disposed to sleep. Abdomen commenced enlarging.

Dec. 14th.—As first seen, abdomen greatly distended ; legs and arms œdematous ; effusion in scrotum ; sensible of a moving fluid in belly upon turning in bed.

℞. Daily purgation, with supertartrate of potassa.
Juniper berry tea as a drink.
Warm bath twice a week.

Feb. 3d, 1842.—Since last date, treatment first adopted regularly persevered with ; rapid improvement succeeded ; excepting slight œdema of legs, all traces of the affection have passed off. Patient expresses himself in perfect health ; appetite good ; digestive functions perfectly performed ; asks to be discharged ; refused, for fear of a recurrence of his disease ; was retained in the wards, from fear of a relapse, until March.

*Remarks.*—In this case, which was one of ordinary liver disease, or cyrrhosis without jaundice, the treatment was perfectly successful ; no alteratives seemed necessary, and the patient was discharged apparently in absolute health. In cases of moderate cyrrhosis the liver may return slowly to its natural condition, but in many individuals it remains indurated, and not sufficiently so to prevent the due performance of its functions, or to again give rise to dropsy from obstruction to the vena portarum; it is always possible that a return of the disease may occur. The patient is placed precisely in the same condition as one labouring under chronic disease of the heart, which may become indolent, after the removal of the accompanying anasarca.

Case 2d.—M—— S——, of muscular development, labourer, admitted December 18th, 1841. Had no other sickness before present attack. Contracted this working upon wharves at Havre de Grace. Had been there only a month before seized by chills and drowsiness. Had three chills. After them dumb agues, sleepiness, general debility. Returned to Philadelphia. Swelled at epigastrium and legs. No jaundice—no treatment.

Dec. 18th.—Entered complaining of chills. Complexion sallow; dark hair and eyes. Abdomen tense; effusion, with fluctuation in the peritoneum; percussion flat; no tenderness but at umbilicus; slight œdema of limbs.

℞. Purged by supertartrate of potassa.
Mass en hydrarg. gr. iij, nocte.
Frictions of iodine ointment, and bandage to abdomen.

Jan. 20th.—Improving; slight ptyalism; blue mass suspended; purge continued. Drinks infusion of buchu leaves and juniper berries. Bandage to abdomen.

31st.—Complexion sallow yet, though vastly improved. Belly softer; no tenderness. Three stools in twenty-four hours; thin and watery. Tongue red, smooth and dry. Pulse seventy-two; soft and regular; skin rather cool; bandage irregularly applied from mistake; sweating abundant towards evening; flush of face.

Feb. 15th.—Much troubled with cough at night; bowels disordered; diarrhœa slight; effusion of abdomen fast diminishing; urine not very abundant; tongue red; papillæ enlarged; appetite good. Pulse 102; not very strong.

℞. Tr. opii camp. ʒi. nocte.

℞. Ol. Ricini ʒij.

Tr. opii, q. h. s. x.

Ter in die.

℞. Iodine frictions and bandage continued.

Feb. 16th.—Much better; diarrhœa diminished to two stools in twenty-four hours; no pain; less distension of abdomen; cough relieved.

25th.—Except a slight cough is still doing well.

March 12th.—Since last date no treatment, except bandage and baths every third day. Convalescence has been rapid. Discharged, cured.

In this patient the general health was extremely deteriorated. The patient had a dull clay-coloured complexion, and was considerably emaciated. The influence of the mercurial action was not very evident; it was at least innocent, which is not always the case with the administration of mercury for chronic diseases of the liver after intermittents. In many such instances the debility increases, and jaundice supervenes during a mercurial course; the medicine then favoring the dropsical effusion and increasing the tendency to ascites. I very rarely prescribe mercury under such circumstances, if the patient be at all anemic, and if the spleen be much enlarged. In these cachectic individuals, it is more frequently mischievous than beneficial. Where the patient is still robust, and the disease of the liver is inflammatory, though in a sub-acute stage, mercury answers a much better purpose. The frictions of iodine ointment were replaced after a few days by applying a muslin cloth spread with iodine ointment under the bandage; this gave rise to much soreness of the skin, which prevented its further use. The application of the iodine is more effectual in favouring the reduction of enlarged viscera, and to some extent of tumours, than of dropsical effusions. The diarrhœa was an accident which followed the continued use of the purgatives; it is always to be dreaded in the treatment of ascites, as incurable softening of the mucous membrane frequently follows diarrhœa originating from this source. As soon as it appears, especially if the tongue be red and dry, purgatives should be immediately discontinued, and the gastro-intestinal irritation removed by soothing remedies.

Case 3d. *Ascites and Jaundice.*—J. M., æt. 29, Irish labourer, admitted Nov. 9th, 1841. Well till last October. Seized with a chill while working upon the Delaware. It did not recur. Feverish for several days afterwards. Dysentery then come upon him. Passed blood from the bowels. Recovering from this, his abdomen swelled; eyes were then jaundiced. Came in complaining of general pains of bones and uneasiness in head.

Nov. 10th.—General jaundiced tint; not much emaciation; effusion of abdomen considerable; urine red, not albuminous.

℞. Calomel gr. iij. at night.

℞. Potass. supt. tart. ʒi.

Pulv. jalap gr. x, in morning.

12th.—Taken two doses of each medicine; feels less oppression; less distended; purged eight or ten times in twenty-four hours; stools large and watery; complexion less jaundiced; tongue moist, rather injected; skin of good temperature; appetite good; thirst moderate; pulse full and soft.

℞. Pulv. jalap gr. xv.
Potass. supt. tart. ʒi.
Calomel discontinued.

16th.—More jaundiced; tongue clean and moist; no tenderness of abdomen; skin moist; pulse soft and regular. Treatment continued.

Dec. 3d.—Jaundiced tint diminished; some cough, with hoarseness.

℞. Leeches No. xx. to larynx.

4th.—Skin slightly tinged; soft, moist, and warm; conjunctiva yellow; hoarseness gone; upwards of a pint of urine passed in twenty-four hours; effusion diminished; four or five stools a-day.

12th.—Condition improved by continued purgation, and alterative effects of mercury; jaundice tint nearly gone, except from eyes.

℞. Acid nit. muriat. q. h. s. x.
Ter in die.

25th.—Acid stopped; purgative continued; feels much better; skin lost its yellow colour; eyes still jaundiced.

Jan. 3d, 1842.—Distension of belly entirely reduced; conjunctiva slightly yellow; all medicines suspended. Patient ready for his discharge.

Retained in wards as an assistant until the 18th of February, to insure his convalescence.

In the treatment of this case, occasional baths were used as in the preceding. A bandage was also applied to the abdomen during the period of the dropsical effusion. The ascites occurred in this case in the acute period of the liver disease with the jaundice; the mercurial purgatives, and cupping several times repeated over the region of the liver, acted extremely well; there was still activity of disease sufficient to justify a directly antiphlogistic course.

The patient was retained in the ward after his discharge from the sick list, for six weeks. There was no dropsical effusion at the time of his leaving the house, and no jaundice, except the faint yellowish tint, which remains in persons who have lived in hot climates. He continued at laborious work for four weeks after his final discharge. At the end of that time, in the middle of this month, (March,) he was taken with increased dropsical swelling, and re-entered the hospital on the 26th of March.

The treatment in these cases of cyrrhosis should not be persevered in for less than three or four months after the removal of the dropsical effusion. In the present case, the patient was retained until he positively required his discharge.

Case 4th.—R. B., æt. 43, intemperate; entered Nov. 18th, 1841, after an attack of delirium tremens. Was well during summer, but has had several attacks of illness including one of dysentery. Was treated two weeks in the cells for delirium tremens before being sent to medical wards. His legs were slightly swelled at the time of his entrance there. It has gradually increased; general anasarca; appetite good. No other symptoms, except dyspnœa on coming up stairs, with palpitations.

℞. Infus. buchu and juniper berries.

℞. Jalap et potass. supt. tart. Bandage to abdomen.

Dec. 3d.—Complexion nearly natural; no cerebral symptoms; tongue slightly coated, moist; no febrile excitement; skin harsh; slight œdema of legs and feet; abdomen distended chiefly with gas; obscure fluctuation at lower portion; slight œdema of cellular tissue. Treatment continued.

Jan. 18th, 1842.—The above treatment was continued until the symptoms gradually yielded; distension of abdomen and œdema of legs have disappeared. Patient discharged.

This case was comparatively a slight one, and the bandage was applied from the time of his entrance until the effusion was almost gone. No return of effusion has taken place. The cases above reported were the only instances of ascites, or of dropsy dependant on liver disease, treated for some months past in one of the divisions of the Philadelphia Hospital. By combining external means with internal remedies, drastic purgatives were dispensed with.

